# “In your face”: The transition from physical to symbolic violence among NBA players

**DOI:** 10.1371/journal.pone.0266875

**Published:** 2022-05-18

**Authors:** Assaf Lev, Gershon Tenenbaum, Omer Eldadi, Topaz Broitman, Jonathan Friedland, Maor Sharabany, Yair Galily

**Affiliations:** 1 Department of Sports Therapy, Ono Academic College, Kiryat Ono, Israel; 2 Sammy Ofer School of Communications, Reichman University, Herzliya, Israel; 3 Sport, Media and Society (SMS) Research Lab, Sammy Ofer School of Communications, Reichman University, Herzliya, Israel; 4 B. Ivcher School of Psychology, Reichman University, Herzliya, Israel; 5 Efi Arazy School of Computer Science, Reichman University, Herzliya, Israel; 6 Adelson School of Entrepreneurship, Reichman University, Herzliya, Israel; Universitat de Valencia, SPAIN

## Abstract

The NBA has undergone formative changes since commissioner David Stern began his tenure. Stern has endeavored to make the NBA a consumer-friendly and marketable league by implementing sanctions on physical violence. This study takes a closer look at Stern’s efforts by examining the interplay between two forms of violence in NBA basketball players on the court: “old fashioned” physical violence (PV) and symbolic violence (SV). Of the 117 NBA finals broadcast for twenty years from 1998 to 2018, a stratified random sample (36 games or 30.8%) of the violations and commentators’ comments were coded, providing a wide perspective on forms of violence over time. The findings reveal that although the number of PV incidents decreased, SV increased starting in 2014, to the extent that SV incidents were more frequent than PV. A thematic analysis of the commentators’ remarks associated with these incidents showed that they support and encourage PV, whereas SV tends to be perceived as harmless and therefore permissible to ignore. Unlike PV, SV is not perceived to be as worthy of media coverage. The relative lack of commentator interest is indicative of the lack of “glamour” of SV in the NBA league. It is suggested that since viewers of professional sports often emulate the players, the increase of SV within the NBA is likely to be mirrored in fans in their everyday lives and in amateur basketball players. Although the rise in SV causes fewer physical injuries in professional basketball players, it nevertheless can cause psychological harm.

## Introduction

Control over assertiveness, aggression, and violence are considered to be the cornerstone of morality [1]. Not surprisingly, these behaviors have been studied in the context of athletics where the name of the game is to dominate one’s opponent and be rewarded for doing so; in some cases, financially. Forceful plays, typically those which are penalized, are forms of aggressive behavior. More extreme aggressive acts (e.g., brawling, attacks on officials/players) are viewed as violent [2]. Within this context, instrumental aggression is defined as behavior directed at a target as a means to an end (e.g., injuring, taking advantage) whereas hostile aggression is considered to target someone who provoked a response. The current study examined aggressive acts committed by players in the National Basketball Association [3] league. NBA commentators label both aggressive and non-aggressive acts as “violence,” whether they aim to harm the opponent or not. In keeping with the media’s definition, “violence” is used interchangeably in what follows to refer to aggressive acts that occur during NBA games and disregards the intention to harm.

As opposed to ‘ordinary’ physically aggressive acts, which can be observed, “symbolic violence” [4] is concealed and framed by the media not only as innocent and harmless, but also as ethical and acceptable. To better understand the origins of symbolic violence, it is crucial to characterize the ways in which those in powerful or dominant positions replicate existing spheres of privilege [5]. Bourdieu [4] argued that symbolic violence “offers an opportunity to grasp the logic of the domination exerted in the name of a symbolic principle known and recognized by the dominant and the dominated—a language (or a pronunciation), a lifestyle (or a way of thinking, speaking, and acting)—and, more generally, a distinctive property, whether emblem or stigma” (pp. 1–2). According to Bourdieu [6], this type of violence is linked to a vast array of norms and values which reproduce domination and inequalities. Lull’s [7] associated concept of “symbolic power” relates to the way an individual or entity can use symbolic violence as a means to sway the course or outcome of events by influencing others’ emotions.

Over the last 20 years, the concept of symbolic violence in the sports literature has become well established [8–11]. Most researchers consider that the conceptual framework of symbolic violence can shed light on the systems of domination and the power relations that create and sustain them. Here, these notions were applied to examine twenty years of TV broadcasts of NBA finals (1998–2018). Within this context, symbolic violence refers to (1) acting while in possession of the ball (e.g., slam dunk to the opponent’s ’face’; i.e., “posterizing), and (2) engaging in symbolic violence while not in possession of the ball (e.g., "trash talk").

In an era when NBA players are sensibly avoiding aggressive moves due to their serious ramifications, the display of aggression and violence has not entirely disappeared. Rather the new version of aggressive and violent behaviors has been converted and adjusted because of fan and media pressure. Both the characteristics and applications of this new symbolic violence are degrading and destructive due to the mass media coverage of the league and the role modeling of star NBA players in many people’s lives, mainly fans and young athletes (e.g., high school and college students).

Exposure to violent media is a risk factor for aggression and violent behavior among viewers, which in turn increases other risk factors such as hostile feelings and thoughts [12–14] and is supported by over six decades of scientific research (for a review, see [15–17]). Exposure to media violence also affects the viewers’ impending behavior as an outcome of social learning (see theoretical models by [18–21]). Hence, repeated exposure to violence through NBA games is likely to influence perceptual, cognitive, and emotional responses and may increase aggressive behavior among viewers.

Although physical aggression and violence have often been denounced by the media in the past two decades [22], symbolic violence may not only “keep the game alive,” but is strongly promoted by the media as enjoyable and humiliating at the same time. Tuchman [23] showed that media content is socially constructed, and journalists as well as commentators often exercise biased judgment on which information to include or exclude in their pieces. Thus, information is vulnerable not only to commentators’ own attitudinal choices, but also to external forces, such as the government, audiences, and advertisers [24]. To capture the intersection of symbolic violence and the role of the media, this study examined contemporary NBA expression of violent actions and the language used by the media to describe them.

### Physical and symbolic violence in basketball: Is there a shift?

Symbolic violence in the NBA dates back some 20 years, but has not emerged in a vacuum. In the last four decades, the NBA has gone through many formative upheavals, most of which relate to its image since David Stern was appointed commissioner in 1984 [25]. When Stern took over, the league was mired in an image crisis [26], which affected the financial health of the franchises. Specifically, 16 out of 23 teams were not profitable in the 1983/84 season [27]. Therefore, to turn things around, Stern’s main concern was to make the NBA a consumer-friendly and highly marketable league [28]. The concepts of consumer-friendliness and marketability are closely correlated with the concept of identification–both with the teams, but mainly with the star players, who are the “face of the league” [29]. However, more importantly much of Stern’s activity involved maintaining a “marketable” image of the players after a series of violent acts led to public condemnations of the league’s inaction [30]. Stern’s reform consisted of changing many of the rules and procedures for drug abuse, dress codes, and violence. Stern’s micromanaging also included bans on entering certain nightclubs and standing at attention with the ‘appropriate’ posture during the National Anthem. One of his key decisions concerned technical fouls. His new rule prohibited players from protesting referee calls based on the rationale that excessive verbal complaints or body language manifesting displeasure increased the likelihood of abuse or violence towards referees [31]. However, his opponents were quick to complain that “the new guidelines threaten to drive emotions out of the game of basketball, even those emotions generally seen as positive, such as camaraderie…” (p. 59). Stern’s regulations dealt primarily with physical violence [32]. This became extremely clear in 2001 when the league instructed referees to exhibit zero tolerance for player violence [3]. The directive ends with a clear statement:

There is absolutely no justification for fighting in an NBA game. The fact that you may feel provoked by another player is not an acceptable excuse. If a player takes it upon himself to retaliate, he can expect to be subject to appropriate penalties. ([3], Comments on the Rules, para. K. Fighting)

The goal of this study was to examine the use of aggression and violence (both physical and symbolic) in the NBA league. To do so, a content analysis of the TV broadcasts of NBA finals over twenty years from 1998 to 2018 was conducted, focusing on both physical and symbolic acts of violence in these games, as well as the language in which these actions were presented in the TV broadcasts. In particular it explored the usage of violent actions since the beginning of the millennium, when the NBA began its zero-tolerance policy for acts of physical violence. The number of incidents of physical aggressive-violent and symbolic violent acts committed in each game were thus tabulated, as well as the importance ascribed to incidents in the broadcasts in terms of the reaction of the anchors.

### Media coverage of sport events

The media coverage of sports events, especially broadcasts such as NBA games which include running patter from commentators, has a significant impact on how the audience perceives these events, since most people watch rather than attend games in person [33]. Since media coverage also affects perceptions of sports and athletes, coverage can also shape the players’ behavior during games [34]. Thus, to study changes in the levels and types of violence used during NBA games, the *attention*, *salience* and *tone* presented within the coverage were examined. The assumption was that in addition to the attention given to the events themselves, a large part of the coverage would be devoted to violence (physical and symbolic), because it fits journalistic norms and the newsworthiness of events. Aggressive and violent occurrences during games raise the level of drama, conflict, personalization, negativity, and entertainment, while providing audio-visual content which the media can use in coverage, and all serve as newsworthiness indicators in today’s media environment [35].

Sports broadcasting is aimed to satisfy viewers. Innovative imaging techniques and camera resolution are employed to enhance the spectators’ media experience. Although viewers are likely to be affected by the visual and auditory features of broadcasts while watching sports on TV, broadcaster commentaries are considered to increase viewer attention and excitement the most [22, 33, 34, 36–38]. For example, ice hockey commentaries that highlight violence were found to provide more entertainment than commentaries that de-emphasized the violence [39]. In a more recent work, when viewers watched tapes of sport events with commentaries that either emphasized or de-emphasized violence, viewers reported greater enjoyment of the scenes of violence the commentators chose to emphasize [22]. In a study where a videotape of a heated Georgetown versus Syracuse men’s college basketball game was viewed, viewers perceived the Syracuse players as being significantly more hostile, in line with the comments expressed by the commentators. Men were more likely than women to enjoy aggression in the game segment, and fans’ perceptions of the opponents’ hostility were as vulnerable to the biased commentary as those of nonfans [38].

Recently, Goldschmied et al. [40] examined the power of broadcasting in framing how spectators construct their interpretations of an observed action [40]. Whereas viewers seated in the stands only see the game play, viewers watching the game on TV see close-ups, replays, and graphics chosen by the producers and can listen to the insights and viewpoints of the sport commentators. In this respect, sport commentators are a unique media source because they blend objective, introspective, and historical components not only to report an event but also to dramatize it [38]. Specifically, the media frames the thoughts and beliefs of the viewers and controls the broadcast by making decisions about the final production [22]. Framing involves the presentation of material, and specifically its organization to consumers [41, 42]. Framing considers selection and salience and is created by determining and highlighting what is being selected. However, framing can also be defined by what is left out, and in many cases, the omissions are more significant or more powerful than the message in the frame. Gitlin [42] defined media framing as “persistent patterns of cognition, interpretation, and presentation of selection, emphasis, and exclusion by which symbol-handlers routinely organize discourse, whether verbal or visual” (p. 7). Thus, framing refers to how information is organized, including what is made to seem important and what is left out or made to seem unimportant, when presented to an audience.

In the current study the contents of the comments expressed by commentators following physical and symbolic acts of violence in the NBA league were coded and categorized considering the assumption that these would have a major effect on the level of empathy and entertainment of those who viewed them. Thus, we aimed at studying the changes in the prevalence of conducting PV and SV acts in the NBA from 1998 to 2018, and the associated comments given to them by the commentators–comments which affect the thoughts, feelings, and behaviors of those who watch and listen to them.

## Method

### Data collection

Of the 117 final NBA TV games broadcast for twenty years from 1998 to2018, a stratified random sample of 42 (35.89%) final games was selected. Specifically, two games were selected randomly in each final series during this time period. Three games were missing and could not be retrieved, and in 6 games, the video and audio quality were too poor for reliable observation, listening, or coding. Thus, the final sample consisted of 36 (30.8%) games which were coded (two games in each final series), providing a wide perspective over time. The video clips originated from public broadcasts (the original quantitative and qualitative data are presented as supplements).

### Data coding, analyses, and emergent themes

To explore the data, a thematic analysis was carried out that consisted of deciphering the texts and identifying explicit rationalizations and their implicit significance [43]. This method served to find common or shared meanings and social experiences [44]. By immersing themselves in the data and carefully reading the transcripts, the researchers were able to determine the relevant themes. Audio recordings of NBA games, specifically the commentators’ remarks on physical and symbolic violence, were transcribed verbatim by a trained research assistant (RA). A qualitative approach using an inductive content analysis [45] where key patterns were extracted from the data was employed. To group the data into meaningful themes, the principal investigator repeatedly read and immersed himself in the transcripts, after which a consensual approach was utilized to analyze the material and resolve differences with fellow researchers to achieve a final aligned version of the coded notes [46]. More specifically, in line with the Garrison et al. [46] approach for coding and reliability checking, the PI coded the entire dataset, identifying components that contained significant information. The codes were then compiled into recurring sets of meaning to generate the main themes. This procedure was iterated until a list of themes was supported by consensus to represent the data. The inter-rater reliability, often termed “investigator triangulation”, a method used to ensure that data are reliable by harnessing intercoder agreement was satisfactory, with a *Kappa* = .93 [47].

### Assurance of quality

To ensure quality and trustworthiness, Tracy’s [48] “eight universal hallmarks for high quality qualitative methods across paradigms” were implemented. Relevant theoretical foundations were applied to achieve the study’s objectives, methodology, and findings. Clear-cut inclusion standards were followed to guarantee that the selected sample was in line with the study’s objectives. This study thus makes a significant theoretical contribution by probing an understudied issue regarding aggression and more specifically types of violence in the NBA league. The data obtained may lead to a better understanding of the manifestation of violence before and after David Stern’s clamp- down on infractions. The findings shed light on the influence of sports commentators on fans worldwide with regards to different forms of violence.

## Results

### Descriptive overview of the data

**Fig 1:**
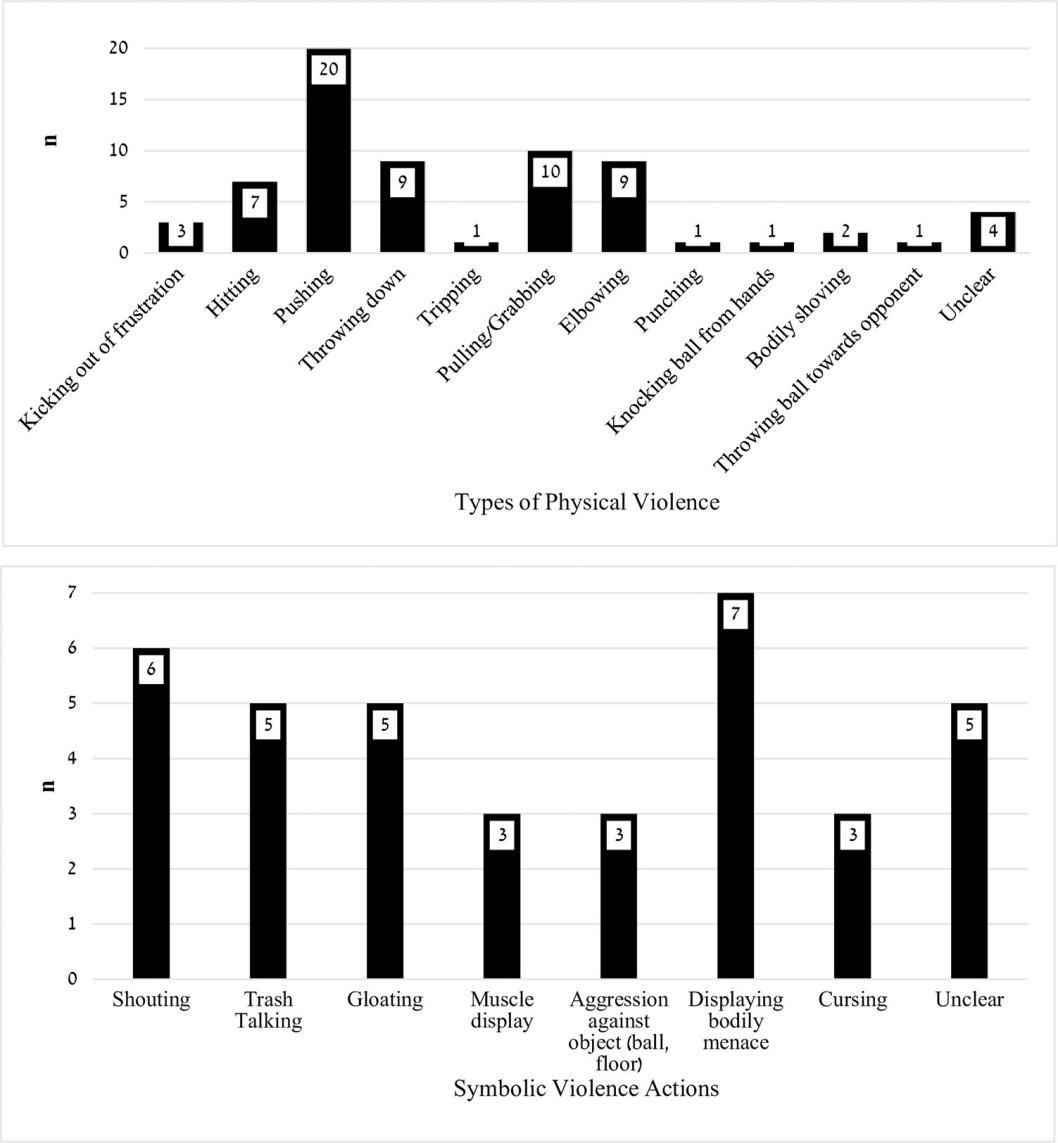
Distribution of physical (upper panel) and symbolic (bottom panel) violent acts in 36 NBA games.

Of the 36 games, 105 violent acts were committed of which 68 (64.76%) were physical and 37 (35.28%) were symbolic. The most frequent physical behaviors were *pushing* (29.41%), *throwing down* and *elbowing* (13.23% each), and *pulling/grabbing* (14.70%). The most frequent symbolic acts were *displaying body menace* (18.92%), *shouting* (16.22%), *trash talking* and *gloating* (13.51% each). The distributions of the violent acts are shown in Fig 1A and 1B.

**Fig 2:**
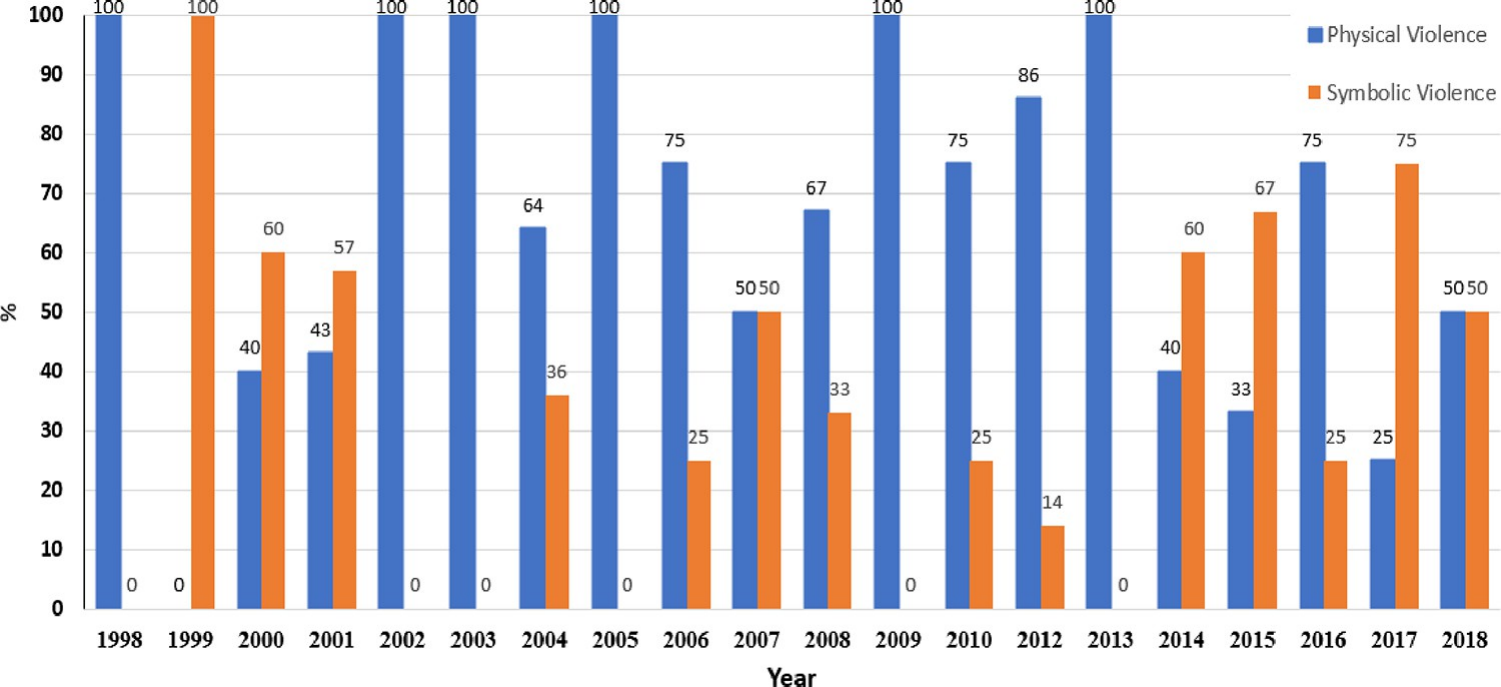
Distribution of physical and symbolic violent acts (in %) from 1998 to 2018 in 36 NBA games.

**Fig 3:**
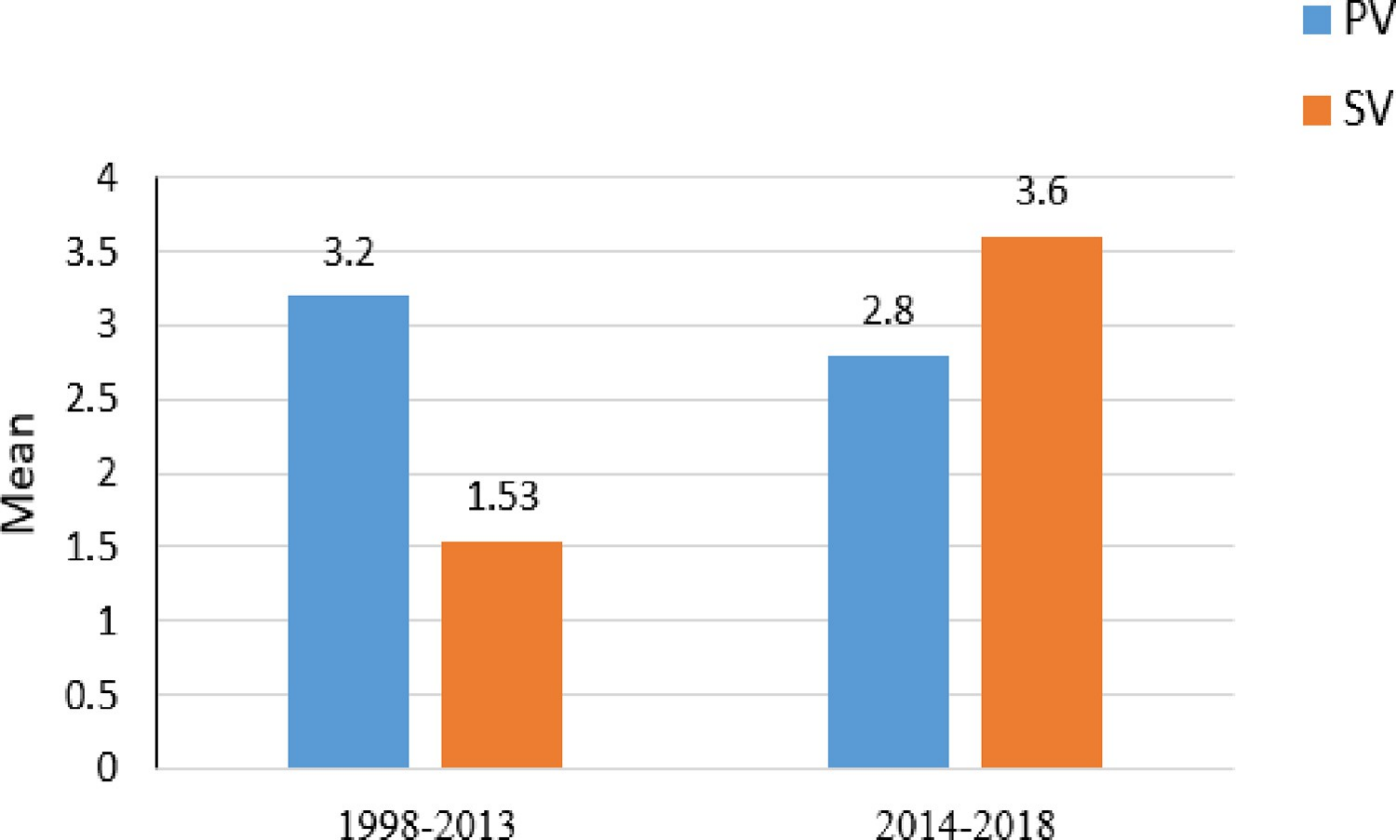
Mean SV and PV during the 1998–2013 and 2014–2018 years.

To contrast the physical and symbolic acts in the games from 1998 to 2018, the number of violent acts in the games was tabulated for each year separately, and the percentages of physical and symbolic acts were calculated (see Fig 2). In general, from 1998 to 2013, physical violent acts were substantially more prevalent than symbolic violent ones (except in 1999, 2000, and 2001). However, from 2014 to 2018, the opposite trend was observed: there were relatively more symbolic violent acts except in 2016 where the prevalence of physical violent acts was higher than symbolic violent acts. The descriptive data thus indicate that the shift in the type of violent acts occurred in 2014, several years after the instigation of the new NBA official policy. To confirm that 2014 was the turning point for the type of violent acts, the mean number of violent acts per year for 1998 to 2013 was compared to the mean for the years 2014–2018. The descriptive data are shown in Fig 3.

Two paired t-tests calculated on the two time intervals indicated that in 1998–2013, significantly more physical than symbolic acts per game were committed (*M* = 3.20, *SD* = 2.30 vs. *M* = 1.53, *SD* = 1.96, respectively), *t*(*df* = 14) = 2.23, *p* < .04. In contrast, in 2014–1018, there was no significant difference between the means of physical and symbolic acts per game although there were more instances of more symbolic violence than physical violence (*M* = 3.60, *SD* = 2.70 vs. *M* = 2.80, *SD* = 2.16, respectively), *t*(*df* = 4) = 0.55, *p* < .61. Two ANOVAs conducted for each violent act separately indicated no significant difference in the mean number of physical violent acts per game in 1998–2013 vs. 2014–2018, *F*(1,18) = 0.12, *p* < .74; however, a strong trend towards an increase in symbolic violent acts from the first to the second time period was evident, *F*(1,18) = 0.12, *p* < .74 (see Fig 3).

### Thematic analysis of the commentators’ media remarks

The data were analyzed in terms of *physical violence* (PV) which players directed toward their opponents during the games, and *symbolic violence* (SV), non-physical acts which players directed toward their opponents. In total there were 55 PV comments and 27 SV comments identified in the corpus. Six themes emerged from the PV data: (1) *Neutral Descriptive Statements*, constituting 18 comments, (2) *Encouragement Statements*, constituting 14 comments, (3) *Condemnation Statements*, constituting 11 comments, (4) *Educational Statements*, constituting 3 comments, (5) *Judgmental Statements*, constituting one comment, and (6) *No Comment*, in 8 segments. Four themes emerged for SV: (1) *Descriptive Statements*, constituting 8 comments, (2) *Encouragement Statements*, constituting 2 comments, (3) *Condemnation Statements*, constituting 2 comments, and (4) *No Comment* in 15 segments.

### Physical violence (PV)

#### Theme 1: Descriptive statements

The first theme that emerged from the comments was *neutral descriptive statement*s. It was evident that while physical violence was taking place on the basketball court, the commentators frequently chose to describe the acts in a descriptive way, with no attempt to criticize or reinforce. Some comments described the physical contact between the players. For example, when a player elbowed his opponent in the head, and both fell to the floor, the commentator stated, “Elbow to the head by Pippen to Stockton. Stockton came and said something to the officials, and he got his technical foul.” In contrast, some comments were more focused on the player’s attitude. For example, when a player attempted to make a basket and his opponent forcefully tackled him to the floor into the crowd, the commentator exclaimed, “A hard foul! … O’Neil is upset, they are trying to hold him down”. Some comments described the nature of the physical act by stressing the NBA rules; for instance, after a player attempted to kick his opponent out of frustration, but failed to make contact, the commentator stated, “According to the rules, that’s a clean play”.

#### Theme 2: Encouragement statements

Whereas some comments were neutral, other NBA commentators encouraged violence during games. For example, commentators described players losing control of their emotions and getting angry and then encouraged the ensuing violence. When a player elbowed his opponent and a flagrant foul was called, the commentator said, “Giving an elbow after the play. I’m glad Karl Malone and Utah [are] finally getting mad”. Another verbal trope employed by commentators to encourage violence was to express joy when players appear to be engaged in a feud, which is often a prelude to physical violence, and to imply that in order to capitalize on their full potential, athletes should use physical violence. In one case where longtime rival players continued to try to make the other fall to the floor, the commentator opined, “I love what I’m seeing, the battle between those two guys… They’re fighting it, giving it everything they’ve got”. In some cases, commentators frame their words as knowledge of the sport while subtly encouraging physical violence. For example, after a player forcefully grabbed his opponent’s jersey to prevent him from scoring, the commentator exclaimed, “Good foul, you know, instead of letting the guy get a dunk, you grab him, and you take the bucket away. Little plays like this can save games”.

#### Theme 3: Condemnation statements

The following theme is indicative of the way acts of physical violence are discouraged by NBA commentators during games. Unlike the encouragement statements, the commentators did not condone violence, but rather commended the referees for their decision to make a certain call based on acts of physical violence committed by players on the court. After an instance where two players were holding onto each other’s jersey to the extent that neither could move, the commentator stated, “They’re going to call a double foul. Both guys were grabbing each other, I think it was an excellent call”. Similarly, after a player elbowed his opponent in the throat, the commentators forcefully condemned the physical act by pinpointing the body part targeted, “Offensive foul! Good call. You see the elbow… right on his throat that’s an easy call”. In some cases, commentators employ condemnation statements implying that the use of physical violence must be curbed because it denigrates the game. For instance, after a player shoved another in the face because he was angry about losing the ball the commentator noted, “Fortunately, Byron Scott quickly calls a timeout to put things back in control”.

#### Theme 4: Educational statements

This theme focuses on comments designed to educate viewers about basketball rules and know-how. For instance, after a player flagrantly fouled his opponent by reaching up and grabbing his neck while the other was jumping for the hoop, the commentator stated, “The definition of a flagrant foul is unnecessary contact, has nothing to do with going to the head or going for the ball”. In another example, after a player was forcefully tackled on his way to the hoop, with the perpetrator not targeting the ball, the commentator educated his viewers by reminding them of an official rule of the game, stating, “Now remember those two guys got taunting fouls against each other in game two… If you don’t try to play the ball usually that’s an automatic flagrant foul”. Unlike the previous examples, in which the commentator aimed to educate the viewers on the rules of the sport, some commentators simply provide general knowledge of the current makeup of the players in the NBA in response to a violent act. After a player forcefully tackled another which resulted in both the latter and a referee falling into the photographers and the crowd, the commentator stated, “Ron Harper is a great sport in the series… he is a competitive guy and he doesn’t want to hurt him”.

#### Theme 5: Judgmental statements

The following theme, which appeared less frequently, categorizes commentators who make derisive statements about players or events on the court when physical violence occurs. For example, after a player tackled his opponent and the latter fell to the floor, seemingly in a staged act, the commentator remarked, “That’s a flop. See, you have to know the guys who flop at this league. And if there is any bit of doubt, you see how he grabs him and falling down. You got to know who is trying to trick you”.

#### Theme 6: No comment

At times, acts of physical violence were met with no reaction. In one circumstance, a player reached for his opponent while the latter was trying to get a rebound, grabbed him and pulled him to the floor with both hands. Although clearly a violation the television commentators had no reaction. In other instances, one player pushed his opponent roughly with his hands and in another case a player used his entire body to shove another. Neither example of physical violence was met with a reaction from the commentator.

### Symbolic violence (SV)

#### Theme 1: Neutral descriptive statements

As found for physical violence, commentators at time made neutral statements towards acts of symbolic violence. While some PV was met with fairly laconic commentary, such as, “plenty of contact there,” “personal foul”, “James is in the follow up”, other instances were addressed more descriptively, and mainly focused on emotions. For example, when a player was fouled by an opponent, the former walked up to the latter and began clapping aggressively and shouting to gloat at his opponent after the referee’s call. The commentator restricted his remarks to a description: “Draymond Green gets overly aggressive, as he bumps up in Smith”. Likewise, when a player was angry when a foul was not called and clenched his fists in rage along with manifestations of aggressive body language towards his opponent, the commentator simply remarked, “He is very, very upset, he thought he is going to have a foul. . . no whistle there”.

#### Theme 2: Encouragement statements

The next theme characterizes the way acts of SV were encouraged by NBA commentators during games. Some encouragement statements described the player himself by associating a complimentary nickname that emphasized his aggressiveness. For example, after a player powerfully slam dunked the ball, pounded his chest and yelled demonstratively, the commentator said, “Tristan Thompson, the beast on the board once again”. Other encouragement statements addressed the play more generally, implying that the SV was needed and should be expected. For example, following an incident on the court, one commentator stated, “Bryant did what he had to…”.

#### Theme 3: Condemnation statements

Condemnations characterize acts of SV that were discouraged by NBA commentators during games. In one instance of SV, after the defense player approached an opponent and bumped his shoulder, the two players started walking down the court towards each other, by staring each other down to intimidate him. The commentator condemned this by stating, “The referees need to get in between these two guys, again things are getting real creepy here”. In a case of “trash talk” between two players after one, who was out of bounds, threw the ball aggressively at the other in the direction of his groin the commentator observed, “Another technical to either one of those guys and they are thrown out so that’s good officiating. You don’t want something to happen in something like that… when the team starts getting in a desperate situation having to win a basketball game, you see all these emotions start to surface”.

#### Theme 4: No comment

Similar to PV cases that elicited no commentator reaction, acts of SV are also often perpetuated, but commentators choose to ignore them despite the blatancy. For example, during a game, a player made a basket and was fouled by an opponent. The former shouted, "and one, yeah motherfucker!”. In another instance, a player was fouled and then stood over the offender looking down at him to show disrespect and to humiliate him. In another example of “trash talking” a player hoped to rile his opponent and start a physical fight. The player’s teammate attempted to pull him away to diffuse the situation, but there was no commentary on this incident. When a player slam dunked his opponent who then raised his fist and shook it at the offending player to intimidate and show disrespect, this again was let pass in silence.

## Discussion

The aim of this study was to examine the interplay between two forms of violence among NBA basketball players on the court: “old fashioned” physical violence (PV) and symbolic violence (SV). The findings revealed that despite David Stern’s harsh strategy of micromanaging the league, and the strict sanctions he imposed to “sterilize” the game of any form of PV since the beginning of the millennium, the latter is still widespread. For example, pushing, pulling/grabbing, elbowing, and hitting still constitute part of the game. However, the findings also clearly indicate a rise of SV in the NBA games since 2014 to the point that the number of SV incidents has overtaken the PV. For instance, displaying bodily menace, shouting, trash talking, and gloating have become a predominant part of the game. Thus overall, in the wake of Stern’s new regulations, the quality of violence has substantially changed. It can be argued that Stern at least partially achieved his goal. Due to multiple restrictions, and more specifically, the threat of sanctions for physical contact, the number of PV incidents on the basketball court has indeed decreased. In other words, watching an NBA game today–either live in a sports arena or on TV–is a different experience than in the pre-Stern era [49]. However, as shown, violence in the NBA has not vanished entirely, and symbolic violence, which is more readily accepted or ignored by NBA commentators has increased. These findings echo Bourdieu’s [4] notion of symbolic violence in that the repetitive display of SV by NBA players results in perceiving it as legitimate. Furthermore, players and commentators tend to accept SV on the court. Thus, symbolic violence has reshaped current manifestations of aggression. Although Stern’s intentions were to rescue the league from its image crisis, his vision failed, at least in terms of eradicating violence per se. Obviously, Stern did not assume that SV would compensate for this new “violence vacuum.” Since SV is not physical the commentators do not feel the need to strongly condemn it, and the drama and “TV- worthy” content is likely to continue and thrive unhindered.

The findings also revealed the significant role of NBA commentators in reporting and dramatizing violence on the court during games. Researchers have long suggested that the media can form or mold thoughts and beliefs and can standardize or control conversation by choosing what to cover and how to portray an event [22]. Commentators blend objective, subjective, and historical components not only to report an event but also to dramatize it [38]. Here, commentators may have influenced both the perceived amount of violence taking place and the audience’s perception of who was the aggressor (ibid). In essence, the framing of the commentary, which tends to control the broadcast by making decisions about the final production [22], can sway viewers to believe something other than what they were seeing.

The most frequent PV and SV theme that emerged from the commentators’ remarks was Neutral Descriptive Statements (n = 18). Here, the commentators described the physical acts as well as the emotional reactions of the players with a neutral statement which could be interpreted as impartial in that they avoided what they saw as embellishment. In other words, during PV the commentators refrained from taking a clear stand. However, in contrast to the Descriptive Statements where PV was described in a “passive” manner, the findings also revealed two frequently used themes that involved taking a stand:—*Encouragement Statements (n = 14)* and *Condemnation Statements (n = 11)*. In the case of encouragement, PV such as pushing and elbowing, which can cause severe injury, elicited positive verbal reactions from the commentators (e.g., “I love what I’m seeing…”). These reactions were often expressed enthusiastically suggesting that commentators lend support and encouragement to PV through their comments and tone. These findings confirm the notion that commentaries that stress violence are regarded as providing more entertainment than commentaries that de-emphasize violence [39].

Because the media plays a significant role in perceptions of sports and athletes, and coverage can also affect the behavior of players throughout the games [34], commentators transmitting a positive message to the audience regarding the use of PV may fuel more violence among athletes on the court, which enhances viewers’ enjoyment [22, 38]. Many viewers consider basketball players to be inspirational figures and role models. Thus, when the players’ violent behaviors on court are received without comments or with minimal consideration, which refrains from condemnation, this behavior becomes socially legitimate and dangerous because it is modeled and can be expressed in real life situations [50] when fans observe the players’ violent behavior and then enact it in other situations [51].

The last theme characterized commentators’ decisions to condemn PV violence. The comments in this theme often commended the referees for their decision to make a certain call based on acts of PV perpetuated by the players on the court. While the frequency of this theme was relatively lower than the previous one, it was nonetheless meaningful. Although the commentators displayed a certain amount of aversion to PV, it should be noted that the comments were often made with a neutral tone. This suggests that while the commentators felt they were expected to condemn PV acts on the court, they did so less willingly and preferred to encourage the violence since this makes for more ‘interesting’ viewing. Thus, commensurate with the Comisky et al [39] position viewers may find more entertainment in broadcasts that emphasize violence.

Similarly, the findings showed that the most frequent comments in response to instances of SV were *Neutral Descriptive (n = 8)*. Like the PV theme, the commentators described SV with “dry” statements, which consisted mainly of an enumeration of the SV and the players’ emotional reaction to them. The theme deserves emphasis because it was by far the most frequent. Specifically, there were only 2 *Encouragement Statement*s and *Condemnation Statements* as compared to their PV counterparts PV. The commentators’ tone was often enthusiastic in their PV Encouragement Statements, whereas they were more neutral or negative in instances of SV. Further, in the Condemnation Statements, the commentators took a neutral or positive tone suggesting that unlike PV, SV was not perceived as attractive for media coverage. The commentators’ relative lack of consideration may be indicative of the lack of “glamour” of SV in the NBA league. Even though SV emerged as occurring more often on the court than PV since 2014, it does not attract the same attention. Because sport commentators draw attention to PV in games to enhance viewers’ enjoyment [22] SV tends to be perceived as unattractive and harmless, but also easy to ignore. Although SV does not result in physical harm to professional basketball players, the psychological damage can be considerable. As viewers of professional sports often emulate the players they see on the court, an increase of SV in the NBA can affect the spectators’ and amateur basketball players’ behaviors in everyday life. Because star NBA players often become major agents of socialization [52], frequent exposure to SV can result in psychologically dangerous consequences.

The findings showed that NBA commentators often egg on the athletes to engage in PV to a greater extent than SV. This may help explain the reason Stern’s endeavor to eradicate physical violence was met with challenges from broadcasters. Sports commentary has a powerful influence on how viewers interpret televised sport [53]. Studies of sporting commentary (e.g., [38, 54]) showed that viewers are highly susceptible to how broadcasters interpret actions and maneuvers in a competition. Their commentaries affect the viewers’ responses and social identity construction [55]. In other words, “The power of images in live sport television often makes us less conscious of the role of language in the work of enhancing and representing events that seem to primarily appeal to the visual senses” [53].

This study of the interplay between physical and symbolic violence can also be applied to other team sports, such as hockey and American football, particularly given the growing condemnation of physical violence in athletic organizations. However, the generalization of the findings must be examined in the light of a number of limitations. First, this study only examined 36 games; a larger sample would provide more representative data. Second, the data collection stopped at 2018, and changes over the course of the past three years may be relevant, especially because of the COVID-19 pandemic. Finally, given that this study focused on male players in the NBA, further research exploring other sports in different cultures/countries and among female athletes is recommended.

## Supporting information

S1 File (DOCX)

S2 File (XLSX)
