# Supplementary material for: “In your face”: The transition from physical to symbolic violence among NBA players
Source: PLoS One. 2022 May 18;17(5):e0266875. doi: 10.1371/journal.pone.0266875 (PMC9116670; doi:10.1371/journal.pone.0266875)
Supplement: S1 File — (DOCX) [file pone.0266875.s001.docx]

| **Quarter; Minute**  Minutes Remaining to Quarter Completion | **Arousal Level** | **Comments’ Tone** | **Commentators’ Comments** | **Game Year** | **Editor** |
| --- | --- | --- | --- | --- | --- |
| Q3,  5:29 | Neutral | Positive | Green goes right at Mozgov, lots of contact but it looks like Mozgov went straight up. and a whistle and a foul  Mozgov going straight up. According to the rules, that's a clean play. Great job. | 2015  Golden State Warriors vs Cleveland Cavaliers  Game 3 | Omer |
| Q2,  08:18 | Neutral | Neutral | Looks like a foul on the other end. Livingstone against Shumpert.  Shumpert and Livingstone are chanting each other. | 2016  Golden State Warriors vs Cleveland Cavaliers  Game 4 |  |
| Q3,  3:06 | Low | Negative | …and then a foul, when Varejao and Kevin love got tangled up.  Love yelling at Crawford (the referee).  The basket will count, and I believe its on Kevin love (the foul).  That's a flop. See, you have to know the guys who flop at this league. And if there is any bit of doubt, you see how he grabs him and falling down.  you got to know who is trying to trick you. |  |  |
| Q3,  2:24 | - | - | No comments |  |  |
| Q4,  7:48 | Neutral | Positive | The coach is right.  No ball movement, a man movement. |  |  |
| Q4,  2:42 | - | - | Unable to attend |  |  |
| Q2,  3:55 | Neutral | Negative | there's a whistle in the backcourt. as Rodman and Malone were entangled, and its number one (foul) on Dennis. Keep your eye on Dennis Rodman, he just grabbed him. not a whole lot there he just grabbed him by the waist. | 1998  Chicago Bulls vs Utah Jazz  Game 5 |  |
| Q2,  2:45 | Neutral | Positive | oh-oh, Malone XXX (give up?) his shot. giving an elbow after the play. I'm glad Karl Malone and Utah finally getting mad. |  |  |
| Q3,  4:45 | Neutral | Neutral | elbow to the head by Pippen to Stockton. Stockton came and said something to the officials, and he got his technical foul. |  |  |
| Q3,  4:20 | Neutral | Neutral | and the whistle blows again. man, can they call it any tighter? the officials are really taking charge here in the third quarter.  Oh yeah, elbow to the face. |  |  |
| Q2  6:53 | Neutral | Neutral | There was whistle on the backcourt, as rodman and Malone were tangled. and its number one do Dennis. Dennis grabbed him by the waist. | 2010  Boston Celtics vs Los Angeles Lakers  Game 2 |  |
| Q4  1:12 | Low | Neutral | Gasol committing the foul, knocking down Perkins. that's the right guy to foul |  |  |
| Q1  1:06 | Neutral | Neutral | Another foul. Popovich is very upset. he thought he had it earlier. | 2005  San Antonio Spurs vs Detroit Pistons  Game 1 |  |
| Q1  6:10 | Low | Positive | Malone and Rodman battling under the board. Malone is going all out as he run the floor, they both kick each other little bit. | 1998  Chicago Bulls vs Utah Jazz  Game 6 |  |
| Q3,  09:05 | High | Positive | Malone in between two red shirts and now he and Rodman tangled up, and it's Malone turn to laugh. |  |  |
| Q3  , 06:08 | High | Positive | Rodman and Malone... Rodman trips Malone. They got to all a flagrant here. They got to call flagrant. I love what I'm seeing the battle between those two guys. they are giving everything they got. |  |  |
| Q3  , 2:21 | Low | Neutral | hard foul! and they going to call it in the act of shooting, and I think that'll discussed whether or not it's a flagrant Call, bringing Steve Javi over to talk about it, whether or not they think it deserves a flagrant foul... that was definitely a flagrant foul right now. the definition of a flagrant foul is unnecessary contact, has nothing to do with going to the head or going for the ball. | 2007  San Antonio Spurs vs Cleveland Cavaliers  Game 1 | Topaz and Maor |
| Q2  , 05:11 | Neutral | Very Positive | Lindsey Hunter was caught, that's why he has a smirk on his face. Veterans have all the tricks, now watch him here, he was grabbing the Jersey and he thought he can get away with it. | 2004  Los Angeles Lakers vs Detroit Pistons  Game 4 |  |
| Q3,  05:37 | Neutral | Very Positive | You know if I am the Lakers I don't sperate... you know when you are a little kid and you kind of push a gut into the other guy, I would have pushed them into Wallace and not separate, because that's a great trade. |  |  |
| Q4,  10:03 | Neutral | Very Positive | Ben Wallace got hugged by walking and there is the foul, but good foul you know instead of let the guy get a dunk, you grab him and you take the bucket away. little plays like this can save games. |  |  |
| Q4,  10:02 | Neutral | Neutral | and there is a battle between them and they going to call a foul. the Lakers bench erupts and their going to call a double foul. both guys were grabbing each other, it think it was an excellent call. |  |  |
| Q2  00:39:39 | High | Negative | and a technical foul on Shaquille O'Neal after the play, he really slump that ball down hard and afterwards he gave Kroshier a shave in his back…. As he gives him a little bump right there. Now Shaquille O'Neal takes a lot of punishments. I am surprised we don't see more of this; he gives him a little shave again. | 2000  Los Angeles Lakers vs Indiana Pacers  Game 6 |  |
| Q3  00:57:02 | High | Positive | and a foul is called. Joh Korfedli, the official pointing at Derek Fisher. Lakers happen overly their foul limit and it looks like Harris is going to the line. | 2002  Los Angeles Lakers vs New Jersey Nets  Game 1 |  |
| Q4  01:11:12 | High | Negative | and he is peerlessly foul by Collins, and you hear the reaction of callings, he had no choice but to prevent the field goal |  |  |
| Q4  01:14:44 | High | Positive | Collins reaching in making contact, and pix up number 4... and then the wild swipe to the ball, he hits Shak in the back of his head, and easy play to call. |  |  |
| Q4  01:18:18 | Hard to attend | Hard to attend | קשה לשמוע בגלל השדרנים הישראליים |  |  |
| Q3  36.6sec | High | Negative | and of the box out by (NAME)… Marcus upset. Too tactical of jersey here. |  |  |
| Q1  11:30 | High | Positive | and a foul is called. Joh Korfedli, the official pointing at Derek Fisher. Lakers happen overly their foul limit and it looks like Harris is going to the line. | 2006  Miami Heat vs Dallas Mavericks  Game 4 |  |
| Q2  09:54 | High | Positive | Offensive foul! good call. You see the elbow... right on his jaw that's an easy call. |  |  |
| Q3  06:30 | High | Very Negative | a hard foul!!! … O'Neil is upset they are trying to hold him down. Now walker gets into Stackhouse space. Dwain Wade kept O'Neil from going after Stackhouse. |  |  |
| Q4,  09:36 | - | - | No comment | 2013  Miami Heat vs San Antonio Spurs  Game 2 |  |
| Q2  00:39:41 | - | - | Cannot be attended | 2002  Los Angeles Lakers vs New Jersey Nets  Game 3 |  |
| Q2,  06:02 | High | Negative | offensive foul…as Westbrook goes into the Haslam hands. They don’t have the typical shot blocking to protect the ring. They do it with their buddies, stepping in. again, now, to me he is lifted up before Haslam got him there. I just would like to see it, being little difficult to get that charge call. | 2012  Miami Heat vs Oklahoma City Thunder  Game 1 |  |
| Q2  31.4 seconds | - | _ | No Comment |  |  |
| Q2,  04:27 | Low | Negative | Foul on Wade on the floor…. He tackles Jams Harden on transition. It looks like he doesn't understand the call | 2012  Miami Heat vs Oklahoma City Thunder  Game 2 |  |
| Q3,  09:39 | Neutral | Negative | That was over aggressive and that's his second foul |  |  |
| Q3,  05:00 | Neutral | Positive | Westbrook looks like he… but he can't get out. When he is getting into the pain good things will eventually happen. He had some. |  |  |
| Q4,  11:27 | High | Negative | offensive foul. Calsion offensive foul, making him looks bad. |  |  |
| Q2  00:52:53 | Low | Neutral | O'Neal… Shaq is having troubling violation, but a foul is called. It's on Tyro Hill! It's his second. | 2001  Los Angeles Lakers vs Philadelphia 76ers  Game 4 |  |
| Q3  02:07:48 | Neutral | Negative | Shaquille O'Neil and that Graver are getting involved. And that foul on Graver, his fourth. |  |  |
| Q4  02:44:13 | - | - | No Comment |  |  |
| Q3, 07:23 | Neutral | Neutral | The crazy thing is that charge certainly on Leonard to LeBron but prior to that there's contact, he should be going to the line for two | 2014  Miami Heat vs San Antonio Spurs  Game 2 |  |
| Q3, 01:52 | Low | Neutral | his energy from the bench has really turned this thing around, Petty Millers has picking up this offensive foul |  |  |
| Q4, 06:43 | Low | Negative | parker goes down hard. parker obviously eats a real pain… I think it's a normal basketball motion, he is just going by him. |  |  |
| Q1, 04:39 | High | Negative | a hard foul | 2014  Miami Heat vs San Antonio Spurs  Game 5 |  |
| Q2  50:56 | High | Very Negative | He is not happy about it. Now remember those two guys got taunting fouls against each other in game two... He made no attempt to play the basketball here, so I'm sure this is going to be a flagrant one, two shots and a ball... If you don't try to play the ball and usually that's an automatic flagrant foul... and they do call it a flagrant. | 2000  Los Angeles Lakers vs Indiana Pacers  Game 3 |  |
| Q3  01:03:23 | - | - | No Comment |  |  |
| Q4,  6:31 | High | Positive | Miller along the base line, he goes tumbling into the role of photographers, and gets cozy with official Bernie Fire, Bernie tried to hold him up and the Reggie was going down, they both went down together, and you see Ron Harper goes over. Ron Harper is a great sport in the series... he is a competitive guy and he doesn't want to hurt him. |  |  |
| Q3,  01:39 | Low | Neutral | A loose ball foul is going to go against Odem. That's actually a great call, Lamar Odem as he was falling down, grabs the arms of the Orlando Magic. While chasing down the loose rebound he realizes he couldn't get it well; if I am not going to get it you guys are coming down with me. | 2009  Los Angeles Lakers vs Orlando Magic  Game 2 |  |
| Q4,  05:59 | - | - | No Comment |  |  |
| Q2,  09:00 | Neutral | Negative | James pushes off Durant, offensive-foul. He didn't like the cal... that's a good call. | 2018  Golden State Warriors vs Cleveland Cavaliers  Game 3 |  |
| Q1  2:40 | High | Negative | He struggled with the flu and emotions last game and you take a look here he didn't like the Ginobli steal and he lets Rose have it in the sternum. That’s what forced the whistle. OH, now he shoves Malik rose, Delaney says what are you doing Kenyon? Just because you didn’t get the call…  Fortunately, Byron Scott quickly calls a timeout to put things back in control.  Losing you mind! Kmart... what is going on? | 2003  New Jersey Nets vs San Antonio Spurs  Game 6 | Jonathan |
| Q2 9:07 | Low | Neutral | Kenyon Martin talking with Dan Crawford, martin was trying to take the ball away from rose after the foul was called, rose held the ball away from him, and you’ll see right here, the foul was called and then Kenyon said “I’ll take that” and then Malik said” no you’re not gonna take that” and they separated the two. |  |  |
| Q1  7:50 | - | - | No Comment | 2008  Los Angeles Lakers vs Boston Celtics  Game 6 |  |
| Q1  7:24 | Low | Negative | Kobe got dragged |  |  |
| Q2  1:23 | Neutral | Neutral | Some pushing and shoving, it seems during the finals they avoid giving double technical, letting the guys show their emotions which has been a good thing.  You see the both guys tangled up. I like the fact  That they understood this coming in. Pierce and Artest fully understood. They talked about having nothing but respect for one another but when they get between the lines they get after it and get after it hard. | 2010  Los Angeles Lakers vs Boston Celtics  Game 7 |  |
| Q1  4:23 | - | - | No Comment | 2004  Los Angeles Lakers vs Detroit Pistons  Game 5 |  |
| Q2  3:36 | Neutral | Negativeת | Lakers are down and now Shaq is on the bench for the rest of the half |  |  |
|  | - | - | Payton was definitely hit in the face by Payton, watch the arm |  |  |
| Q1  3:50 | - | - | Can't hear English commentary – but doesn’t sound like they said much. | 2016  Golden State Warriors vs Cleveland Cavaliers  Game 2 |  |

|  |  |  |  |  | **Editor** |
| --- | --- | --- | --- | --- | --- |
| Q4,  29 sec left | - | - | No Comments | 2015  Golden State Warriors vs Cleveland Cavaliers  Game 3 | Omer |
| Q4,  16.8 sec left | - | - | No Comments |  |  |
| Q1,  2:07 | Neutral | Positive | Tristan Thompson the beast on the board once again | 2016  Golden State Warriors vs Cleveland Cavaliers  Game 4 |  |
| - | Neutral | Neutral | Plenty of contact there |  |  |
| Q1  10:03 | Neutral | Neutral | Draymond Green gets overly aggressive, as he bumps up in Smith. | 2017  Golden State Warriors vs Cleveland Cavaliers  Game 3 |  |
| Q2,  11:47 | Neutral | Neutral | Personal Foul. |  |  |
| Q2  4:40 | - | - | Unclear |  |  |
| Q3 | Low | Positive | The ability to get the contact and finish with the opposite hand. |  |  |
| Q2  11:39 | No Comments | No Comments | No Comments | 2007  San Antonio Spurs vs Cleveland Cavaliers  Game 1 | Topaz and Maor |
| Q1  04:20 | No Comments | No Comments | No Comments | 2004  Los Angeles Lakers vs Detroit Pistons  Game 4 |  |
| Q3  02:11:19 | High | Negative | לא שומעים כי השדרנים הישראליים נכנסים | 2000  Los Angeles Lakers vs Indiana Pacers  Game 6 |  |
| Q4  02:11:19 | - | - | Cannot attend |  |  |
| Q2 04:50 | No Comments | No Comments | No Comments | 2006  Miami Heat vs Dallas Mavericks  Game 4 |  |
| Q1  00:18:29 | High | Positive | he is very very upset, he thought he is gonna have foul... no whistle there | 1999  San Antonio Spurs vs New York Knicks  Game 5 |  |
| Q2  00:29:59 | Low | Negative | none of this little touch fouls, if you are going to create contact make sure it's a good foul let's not pick up your third on anything cheap. |  |  |
| Q2  00:39:05 | No Comments | No Comments | No Comments |  |  |
| Q4  01:11:50 | No Comments | No Comments | No Comments |  |  |
| Q2,  04:47 | No Comments | No Comments | No Comments | 2012  Miami Heat vs Oklahoma City Thunder  Game 1 |  |
| Q2,  04:04 | No Comments | No Comments | No Comments |  |  |
| Q1  00:37:02 | Neutral | Neutral | Allen Iverson just goes one of his first six | 2001  Los Angeles Lakers vs Philadelphia 76ers  Game 4 |  |
| Q2  01:02:01 | Neutral | Neutral | And a foul on Iverson. Iverson is very upset and actually launch down the court just to get away from the officials. |  |  |
| Q3  01:52:26 | - | - | No Comments |  |  |
| Q4  02:42:14 | - | - | No Comments |  |  |
| Q1,  08:41 | High | Positive | James is in the follow up | 2014  Miami Heat vs San Antonio Spurs  Game 5 |  |
| Q1  24:10 | - | - | No Comments | 2000  Los Angeles Lakers vs Indiana Pacers  Game 3 |  |
| Q2  47:11 | High | Neutral | guys get a little "cheapy" and that what's happens in playoff serious, the longer it goes you get aggravated to see this guy over and over, he cracks you in game two, you are going to get him back in game 3, the ball goes a little loose, they wrestle around over there for it... that's what happens in long Series. |  |  |
| Q3  01:03:32 | High | Negative Very | the referees need to get in between these two guys, again things getting real cheapy here... I can read Glan Rice's lips, he say, "come on there is nothing here, come on let's play" and Davis is pressing the issue. |  |  |
| Q3  01:15:33 | Neutral | Positive | Good referee in there by Hubert. It was over Ron Harper and Mark Jackson got technical early in the game. another technical to either one of those guys and they are thrown out so that's good officiating. you don't want something to happen in something like that... when its' team starts getting in a desperate situation having to win a basketball game, you see all these emotions stat to surface. |  |  |
| Q1,  04:49 | Neutral | Positive | And a foul on Draymond Green. Green walking away from the officials. There is a lot of contact here. | 2018  Golden State Warriors vs Cleveland Cavaliers  Game 3 |  |
| Q2  9:57 | High | Negative | Bryant did what he had to, to better negotiate that screen.  and a technical foul on doc rivers! Joe Crawford has warned the coaches during the previous break to stop the complaining  I think he was complaining about the way kobe Bryant negotiated that last screen  Bryant did what he had to, to better negotiate that screen, Doc Rivers didn’t like it. | 2008  Los Angeles Lakers vs Boston Celtics  Game 6 | Jonathan |
| Q3,  10:25 | - | - | No Comments | 2010  Los Angeles Lakers vs Boston Celtics |  |
| Q2.  4:15 | - | - | No Comments | 2004  Los Angeles Lakers vs Detroit Pistons  Game 5 |  |
